# Performance of contrast-enhanced cone-beam breast CT to predict nipple–areolar complex involvement in early-stage breast cancer

**DOI:** 10.1007/s00330-025-11787-8

**Published:** 2025-07-01

**Authors:** Jie Huang, Ni He, Jiao Li, Jieting Chen, Canyu Guan, Yaopan Wu, Qianyi Lu

**Affiliations:** Department of Radiology, State Key Laboratory of Oncology in South China, Guangdong Provincial Clinical Research Center for Cancer, Sun Yat-Sen University Cancer Center, Guangzhou, P. R. China

**Keywords:** Nipple, Breast neoplasms, Cone beam computed tomography, Mastectomy

## Abstract

**Objectives:**

To evaluate the diagnostic performance of preoperative contrast-enhanced cone-beam breast CT (CE-CBBCT) and identify significant predictors of nipple–areolar complex (NAC) involvement in early-stage breast cancer patients.

**Materials and methods:**

This retrospective study included 641 breast cancer cases from 631 patients at Sun Yat-sen University Cancer Center (2019.3–2021.3). From these, 182 cases were selected after one-by-one matching with the NAC involvement group using the propensity score matching method. Two radiologists independently assessed CE-CBBCT imaging factors in 182 cases. Diagnostic performance indices were analyzed, and predictors of NAC involvement in breast cancer were identified using logistic regression analyses.

**Results:**

The 182 matched cases were females with a median age of 50 (interquartile range, 44–55; range, 25–81 years). Interobserver agreement regarding CBBCT prediction by two radiologists was relatively substantial (κ = 0.730). The accuracy of radiologists in predicting NAC involvement in CE-CBBCT was 83.52% (152/182), with a sensitivity of 96.70% (88/91), specificity of 70.33% (64/91), negative predictive value of 95.52% (64/67), and positive predictive value (PPV) of 76.52% (88/115). On CE-CBBCT, asymmetric NAC enhancement (odds ratio, 5.279; *p* = 0.001) and TNE (tumor–nipple enhancement) within 2 cm of the NAC (odds ratio, 4.184; *p* = 0.02) were significant predictors of NAC involvement. When asymmetric NAC enhancement and TNE extending to the NAC were present, the PPV was 82.35% (56/68).

**Conclusions:**

CE-CBBCT is a safe and non-invasive modality with comparably high accuracy for the preoperative diagnosis of NAC involvement. Asymmetric NAC enhancement and TNE within 2 cm of the NAC performed well in predicting NAC involvement.

**Key Points:**

***Question*** *Several imaging modalities have been studied to preoperatively evaluate NAC involvement, but CE-CBBCT's performance was unknown*.

***Findings*** *CE-CBBCT showed high accuracy in diagnosing NAC involvement. Asymmetric NAC enhancement and tumor–nipple enhancement within 2* *cm of the NAC were independent predictors of NAC involvement*.

***Clinical relevance*** *CE-CBBCT can serve as a safe, non-invasive modality to diagnose NAC involvement preoperatively and help to identify candidates for nipple-sparing mastectomy*.

**Graphical Abstract:**

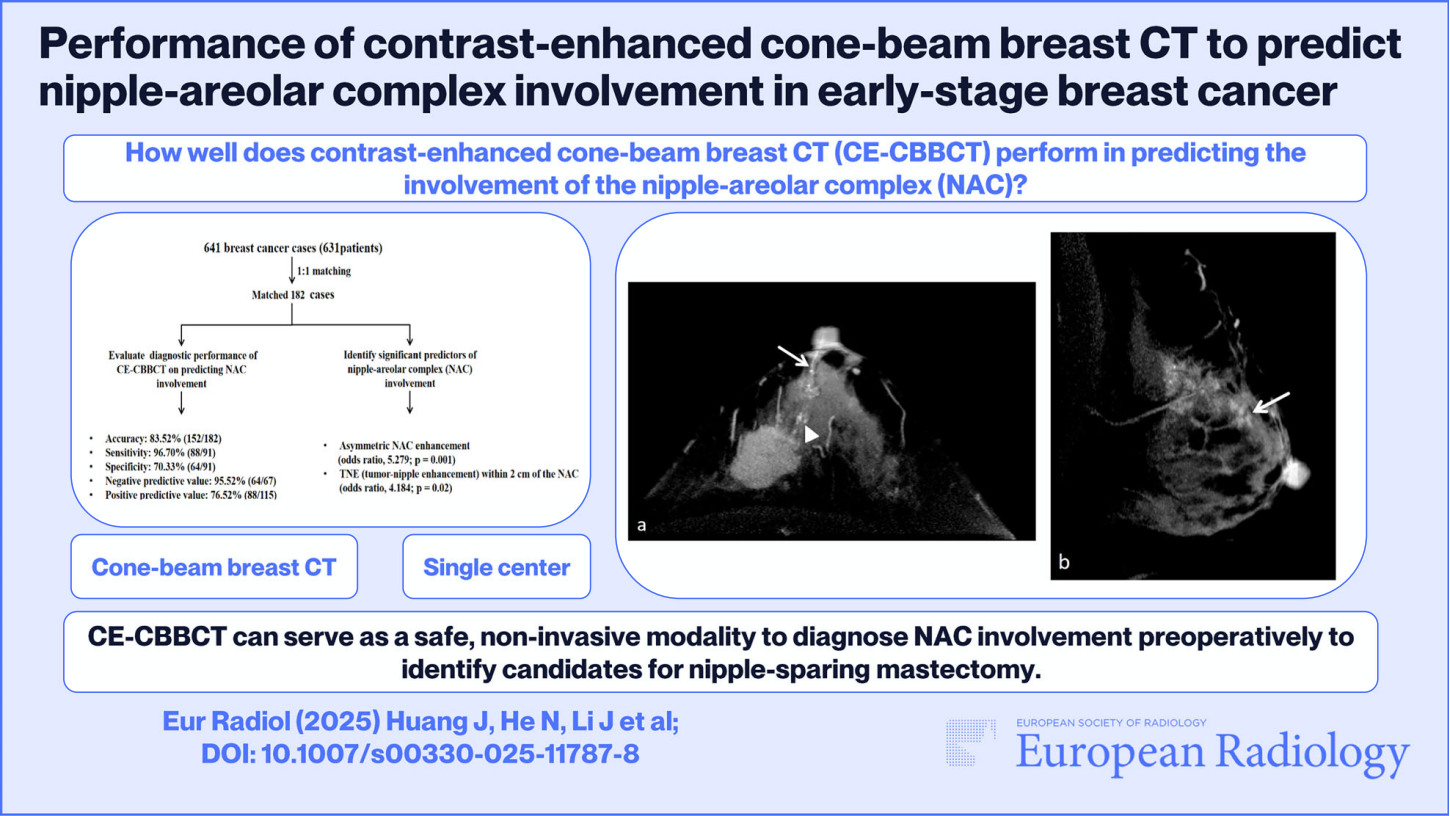

## Introduction

Nipple-sparing mastectomy (NSM), which removes the gland but preserves the nipple–areola complex (NAC) and skin, is increasingly used as a conservative surgical approach for breast cancer to satisfy the psychological needs of patients [1–3]. However, previous studies have reported that the incidence of NAC involvement ranges from 8% to 27.7% [4–10], and that clinical and radiological NAC involvement are contraindications for NSM [3]. Therefore, it is necessary to accurately evaluate preoperative NAC involvement. Several studies have been conducted to predict NAC involvement using mammography, breast ultrasound, dynamic enhanced magnetic resonance, and 18F-FDG PET/CT [9, 11–15] and the accuracy ranged from 80.8% to 87.4%, with a sensitivity of 60.5% to 92% [9, 12, 14, 15]. MRI features significantly correlated with NAC, such as enhancement between the index lesion and the nipple, abnormal nipple enhancement, tumor–nipple distance (TND), nipple retraction, and thickening of the NAC [4, 6, 13, 15–19].

Contrast-enhanced cone-beam breast CT (CE-CBBCT), which provides images with no compression, removal of tissue overlaps, rapid acquisition, and available simultaneous assessment of microcalcifications and contrast enhancement, has been approved by Chinese National Medical Products Administration, the U.S. FDA, and the European Union, and is increasingly used for breast imaging [20]. Many studies have been conducted on CBBCT to diagnose breast diseases, predict the prognostic stage of breast cancer, predict axillary lymph node metastasis of breast cancer, assess residual tumors after neoadjuvant chemotherapy for breast cancer, and guide breast vacuum-assisted biopsy [20–27]. However, few studies have investigated its value in predicting NAC involvement, and data on the accuracy of CE-CBBCT for evaluating NAC involvement are limited.

Therefore, in this study, we aimed to evaluate the diagnostic performance of preoperative CE-CBBCT and identify significant predictors of NAC involvement in patients with early-stage breast cancer.

## Materials and methods

### Study population

The Institutional Review Board of Sun Yat-sen University Cancer Center approved this retrospective study and waived the requirement for informed patient consent. Since CBBCT received approval for clinical use from Chinese National Medical Products Administration (registration no. 20153062052), the U.S. FDA (PMA no. P130025), and the European Union (CE Mark Certificate Number 1084CE), and after Sun Yat-sen University Cancer Center acquired the CBBCT equipment in 2012, it has been integrated into routine clinical practice. The decision to perform cone-beam breast CT (CBBCT) for additional lesion assessment is made by clinicians based on clinical indications (e.g., contraindications for MRI, calcification assessment, etc.), individual considerations, and so on.

**Fig. 1:**
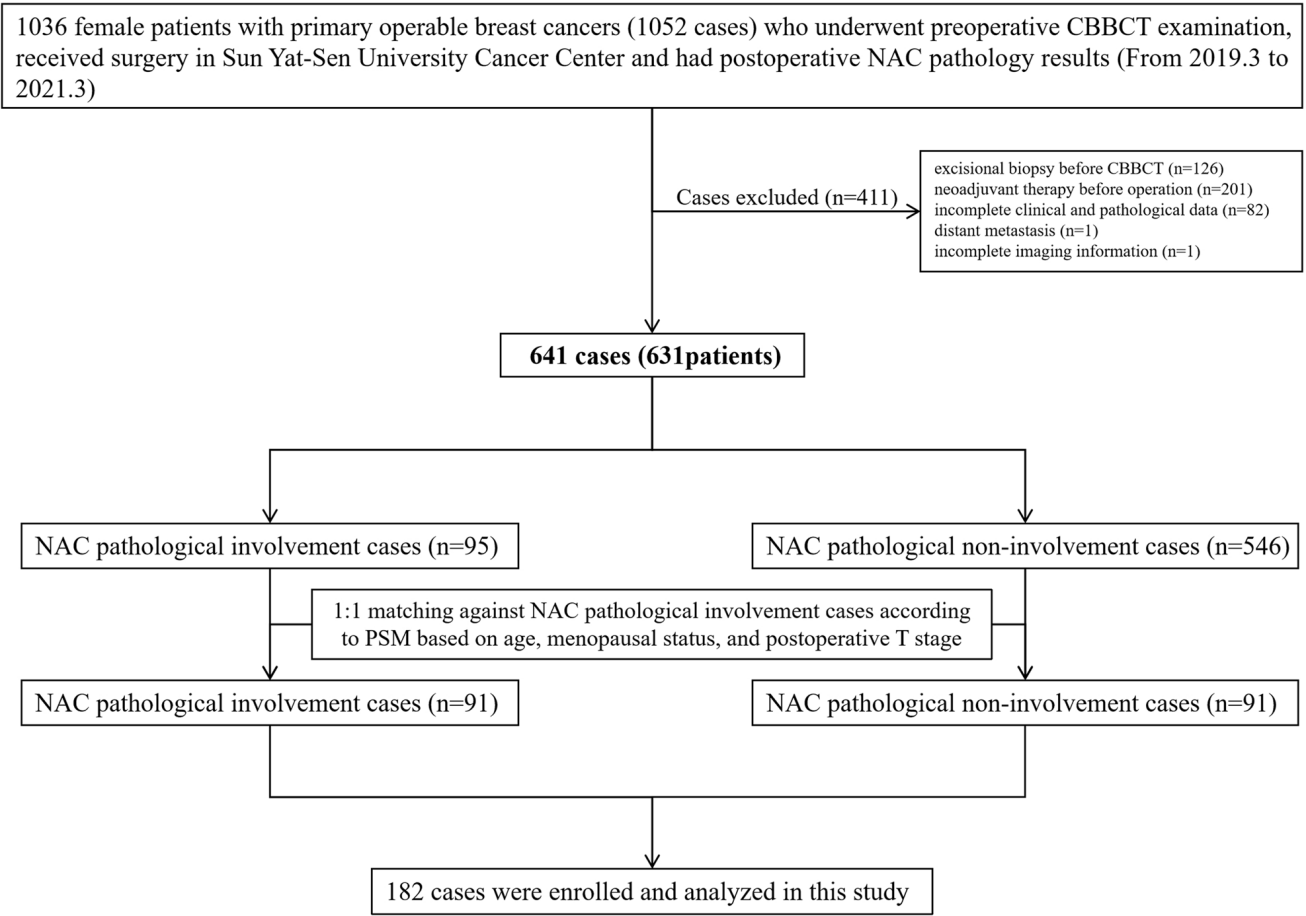
Flowchart of patient selection. CBBCT, cone-beam breast CT; NAC, nipple–areolar complex; PSM, propensity score matching

Between March 2019 and March 2021, 1036 female patients with primary operable breast cancers (1052 cases) who underwent preoperative CBBCT examination, received surgery in Sun Yat-Sen University Cancer Center, and had postoperative NAC pathology results, were selected from the hospital’s pathology database. The exclusion criteria encompassed patients who underwent excisional biopsy surgery before CBBCT, received neoadjuvant therapy before operation, had incomplete clinical and pathological data, had distant metastasis, or had incomplete imaging information. This selection process enrolled 641 breast cancer cases in 631 patients, including 95 pathological NAC involvement cases and 546 pathological NAC non-involvement cases. In this population, one-by-one propensity score matching against the pathological NAC involvement group was conducted based on age, menopausal status, and postoperative T stage. This resulted in 91 pathological NAC involvement cases being matched to 91 pathological non-involvement cases, yielding 182 cases for inclusion in this study (Fig. 1).

### CBBCT protocol

All patients underwent CBBCT (KBCT1000, Koning Corporation), which was approved by the Chinese National Medical Products Administration, the U.S. FDA, and the European Union. During CBBCT scanning, patients were positioned in a prone posture with elevated arms to ensure the natural hanging of the breast within the scanning field. After positioning, non-contrast-enhanced CBBCT was performed. Subsequently, two-phase enhanced scanning was performed on the affected breast without repositioning using identical scanning parameters at intervals of 60 s and 110 s, respectively, where the iodine contrast agent was intravenously injected using a high-pressure injector at a 1–2 mL/kg concentration and a speed of 2.0–3.0 mL/s. A single-phase enhanced scan was performed on the contralateral breast after changing its position, following the affected breast's enhanced scanning. The scanning parameters were as follows: constant X-ray tube voltage of 49 kVp and automatic tube current adjustment based on breast density and size (50–160 mA). For a single continuous acquisition scan, 300 2D projection images were acquired by rotating the X-ray tube and the X-ray flat-panel detector 360° around the breast in 10 s. The X-ray pulses were of 8 ms duration at a frequency of 30 Hz [28, 29]. Three-dimensional breast image reconstruction with a voxel size of 0.273 mm^3^ was achieved using a soft-tissue filter in standard mode.

### Clinical, pathologic, and imaging analyses

Clinicopathological information, including age, menopausal status, histologic subtype, nuclear grade, tumor stage, postoperative T stage, postoperative N stage, estrogen receptor (ER), progesterone receptor (PR), human epidermal growth factor receptor (HER-2) expression, Ki-67 expression, molecular subtype, lymphovascular invasion, and NAC involvement status, were collected retrospectively. ER and PR positivity was defined as at least 1% of the tumor nuclei being positive. HER-2 amplification was defined as 3 + based on immunohistochemistry or gene amplification by in situ hybridization.

Two radiologists, one with 3 years of diagnostic experience in CBBCT and the other with 4 years in CBBCT, independently evaluated imaging features, blinded to the final pathological outcome of NAC involvement. Before the evaluation, both radiologists reviewed the relevant literature and underwent training to ensure consistency in interpreting imaging features. For categorical variables, each observer independently assessed the cases, and an agreement was obtained by consensus or by a more experienced radiologist in cases of discordance. A single radiologist directly measured the objective numerical variables. The imaging features assessed in CBBCT included asymmetric NAC enhancement (yes/no), nipple retraction (yes/no), periareolar skin thickening (yes/no), suspicious calcification within 2 cm (yes/no), multicentricity/multifocality of tumor (yes/no), tumor location (central area only/also involving peripheral area), overall size, largest diameter, TND (distance from the anterior edge of the tumor to the nipple base), the morphology of the lesion enhancement (only mass/including non-mass-enhancement), and tumor–nipple enhancement (TNE) within 2 cm of the NAC (no/enhancement extending to the NAC/enhancement non-extending to the NAC but within 2 cm). Radiologists also provided overall predictions of NAC involvement (involvement/non-involvement).

**Fig. 2:**
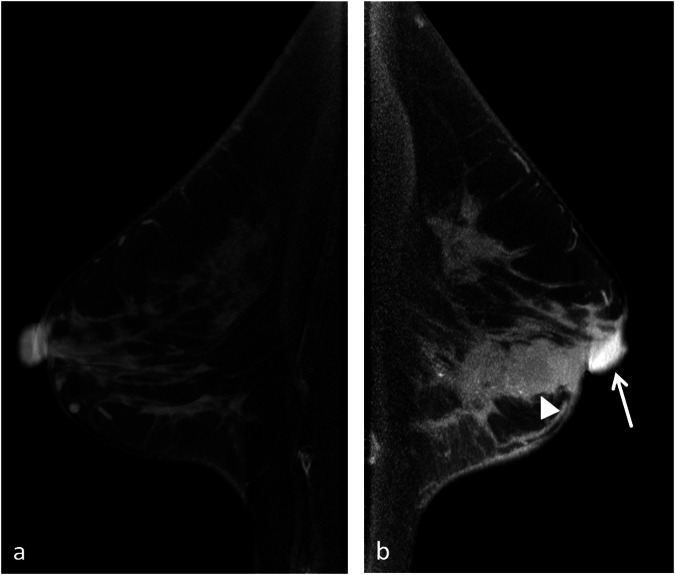
CE-CBBCT of a 58-year-old patient with invasive ductal carcinoma (arrowhead) in the left breast (**b**). In this patient, there was a conspicuous enhancement on the left nipple (arrow), while there was no enhancement on the right nipple (**a**), which was a typical example of asymmetric NAC enhancement. In addition, nipple retraction and periareolar skin thickening were observed. NAC involvement on the left nipple was confirmed by postoperative pathology. CE-CBBCT, contrast-enhanced cone-beam breast CT; NAC, nipple–areolar complex

**Fig. 3:**
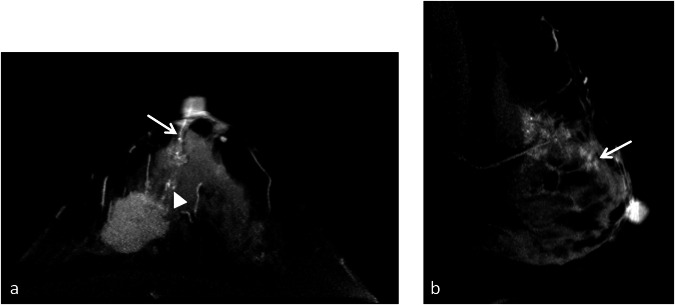
Examples of TNE within 2 cm of the NAC. **a** A 40-year-old patient with invasive ductal carcinoma in the left breast. An axial CE-CBBCT image showed examples of TNE extending to the NAC (arrow) and suspicious calcification within 2 cm (arrowhead). A mastectomy was performed, and NAC involvement was detected in pathology. **b** A 50-year-old patient with invasive ductal carcinoma in the left breast. A sagittal CE-CBBCT image showed an example of TNE non-extending to the NAC but within 2 cm (arrow). A mastectomy was performed, and NAC involvement was not detected. NAC, nipple–areolar complex; CE-CBBCT, contrast-enhanced cone-beam breast CT; TNE, tumor–nipple enhancement.

Multicentricity/multifocality of the tumor was defined as multiple lesions in more than two quadrants or within one quadrant. For such lesions, the total size of all lesions within the breast and the largest diameter of the maximum lesion were recorded, and the overall size and largest diameter were named, respectively. The central area of the tumor was defined as the cylindrical breast tissue extending from the dermis behind the NAC to the pectoral fascia. The TND was measured in the sagittal plane using two methods: if the anterior edge of the tumor and the nipple were in the same plane, the distance was directly measured; if in different planes, TND was obtained from the sagittal Maximum Intensity Projection image of CBBCT. Asymmetric NAC enhancement was defined as a conspicuous difference in the enhanced area between the observed and contralateral nipple [15, 17, 30] (Fig. 2). In other words, when the contralateral nipple exhibited a similar enhancement, asymmetric NAC enhancement was considered absent. Seven patients who underwent contralateral mastectomy were excluded from the regression analysis of this variable. Nipple retraction refers to the focal inward pulling of a portion of the nipple, which is typically asymmetric. Suspicious calcification within 2 cm was assessed based on its shape, distribution, and relationship with the tumor. TNE within 2 cm of the NAC was considered ‘yes’ if there was an enhancement in the direction of the NAC from the tumor, and its anterior edge was less than 2 cm away from the base of the NAC but did not include enhancement of the tumor itself within 2 cm. We further categorized this feature as TNE extending to the NAC or TNE non-extending to the NAC but within 2 cm [4, 6, 16, 31] (Fig. 3).

### Statistical analysis

The diagnostic performance of CE-CBBCT was evaluated in terms of sensitivity, specificity, positive predictive value (PPV), negative predictive value (NPV), and diagnostic accuracy. Interobserver agreement on NAC involvement between two observers was assessed by calculating κ values. Pearson’s chi-square test was used to evaluate the association between NAC involvement and clinicopathological imaging factors. Factors that showed a significant association with the outcome were assessed using univariate and multivariate analyses with a logistic regression model with enter modeling to establish a predictive model, which was then evaluated using receiver operating characteristic (ROC) curves. A more detailed analysis of significant feature combinations was conducted for different combinations of features concerning sensitivity, specificity, NPV, PPV, accuracy, and area under the curve (AUC) calculated using ROC.

Data analyses were performed using IBM SPSS Statistics (version 25.0; IBM Corp.). A *p*-value of less than 0.05 was considered statistically significant for all analyses. The κ value was classified as slight (< 0.20), fair (0.21–0.40), moderate (0.41–0.60), substantial (0.61–0.80), and almost perfect (0.81–1.0) [17].

## Results

In our original population of 631 patients (641 breast cancer cases), all patients were female with the median age of 51 years (interquartile range [IQR], 45–58; range, 25–81 years), and approximately 14.82% (95/641) of our original study population had pathologically confirmed NAC involvement. After matching, a later statistical study included the median age of 50 years (IQR, 44–55; range, 25–81 years) and 182 cases (91 pathological NAC involvement cases against 91 pathological NAC non-involvement cases). Interobserver agreement regarding the CBBCT prediction by two radiologists was substantial, with a κ value of 0.730.

One hundred fifty-two out of 182 cases were accurately predicted by the radiologists compared to the pathology results, with 88 accurately predicted as having NAC involvement and 64 as not. The accuracy of predicting NAC involvement in CE-CBBCT by radiologists was 83.52% (152/182), with a sensitivity of 96.70% (88/91), specificity of 70.33% (64/91), NPV of 95.52% (64/67), and PPV of 76.52% (88/115) (Table 1).

**Table 1:** Correlation between predictive NAC involvement by radiologists and pathologic results

		Pathological NAC involvement		
		Non-involvement	Involvement	Total
Predictive NAC involvement	Non-involvement	64 (95.5%)	3 (4.5%)	67 (100.0%)
	Involvement	27 (23.5%)	88 (76.5%)	115 (100.0%)
		91 (50.0%)	91 (50.0%)	182 (100.0%)

Sensitivity 96.70%, Specificity 70.33%, PPV 76.52%, NPV 95.52%, Accuracy 83.52%

*NAC* nipple–areolar complex, *PPV* positive predictive value, *NPV* negative predictive value

On univariate analysis by chi-square testing, postoperative N stage (*p* = 0.02), HER-2 status (*p* = 0.007), asymmetric NAC enhancement (*p* < 0.001), nipple retraction (*p* < 0.001), periareolar skin thickening (*p* < 0.001), suspicious calcification within 2 cm (*p* < 0.001), multicentric/multifocal lesion (*p* < 0.001), overall size (*p* < 0.001), TND (*p* < 0.001), morphology of the lesion (*p* < 0.001), and TNE within 2 cm of the NAC (*p* < 0.001) were significantly different between two groups (Table 2).

**Table 2:** Clinicopathologic factors and radiologic features in CBBCT images in patients with or without NAC involvement based on pathological diagnosis

		Pathological NAC involvement		*p* value
		Non-involvement (*N* = 91)	Involvement (*N* = 91)	
**Clinicopathologic factors**				
Postoperative *N* stage				0.02
	0	56 (61.5%)	44 (48.4%)	
	1	24 (26.4%)	18 (19.8%)	
	2	5 (5.5%)	14 (15.4%)	
	3	6 (6.6%)	15 (16.5%)	
HER-2 status				0.007
	Not amplified	59 (64.8%)	41 (45.1%)	
	Amplified	32 (35.2%)	50 (54.9%)	
**Radiologic features**				
Asymmetric NAC enhancement^*^				< 0.001
	No	67 (77.9%)	20 (22.5%)	
	Yes	19 (22.1%)	69 (77.5%)	
Nipple retraction				< 0.001
	No	79 (86.8%)	52 (57.1%)	
	Yes	12 (13.2%)	39 (42.9%)	
Periareolar skin thickening				< 0.001
	No	76 (83.5%)	37 (40.7%)	
	Yes	15 (16.5%)	54 (59.3%)	
Suspicious calcification within 2 cm				< 0.001
	No	71 (78.0%)	46 (50.5%)	
	Yes	20 (22.0%)	45 (49.5%)	
Multicentric/multifocal lesion				< 0.001
	No	45 (49.5%)	12 (13.2%)	
	Yes	46 (50.5%)	79 (86.8%)	
Overall size				< 0.001
	≤ 2 cm	25 (27.5%)	4 (4.4%)	
	> 2 cm	66 (72.5%)	87 (95.6%)	
TND				< 0.001
	> 1 cm	71 (78.0%)	30 (33.0%)	
	≤ 1 cm	20 (22.0%)	61 (67.0%)	
Morphology of the lesion				< 0.001
	Only mass	63 (69.2%)	30 (33.0%)	
	Including NME	28 (30.8%)	61 (67.0%)	
TNE within 2 cm of the NAC				< 0.001
	No	63 (69.2%)	9 (9.9%)	
	TNE extending to the NAC	17 (18.7%)	67 (73.6%)	
	TNE non-extending to the NAC but within 2 cm	11 (12.1%)	15 (16.5%)	

Additional details are provided in the Electronic Supplementary Material. HER-2 amplification was defined as 3 + using immunohistochemistry or gene amplification by in situ hybridization

*NAC* nipple–areolar complex, *HER-2* human epidermal growth factor receptor-2, *TND* tumor–nipple distance, *NME* non-mass enhancement, *TNE* tumor–nipple enhancement

* Missing values

Among the 182 cases, after excluding seven cases due to failure to evaluate the contralateral nipple, 89 cases showed NAC involvement pathologically, and 86 cases showed NAC non-involvement among the remaining 175 cases. We further conducted a logistic regression analysis on this population’s significant factors mentioned above. We found that only asymmetric NAC enhancement (*p* = 0.001) and TNE within 2 cm of the NAC (*p* = 0.02) remained significant in multivariate analysis, with odds ratios (ORs) and 95% confidence intervals (CIs) of 5.279 (95% CI: 2.005–13.898) and 4.184 (95% CI: 1.260–13.898), respectively (Table 3). The AUC of the regression model was 0.882.

**Table 3:** Univariate and multivariate analysis of NAC involvement

Variable	Univariate				Multivariate			
		95% CI				95% CI		
	OR	Lower	Upper	*p* value	OR	Lower	Upper	*p* value
HER-2 status (not amplified vs amplified)	2.185	1.189	4.016	0.01	2.207	0.928	5.248	0.07
Multicentric/multifocal lesion (no vs yes)	5.846	2.787	12.266	< 0.001	2.872	0.790	10.441	0.11
Asymmetric NAC enhancement (no vs yes)	12.166	5.968	24.800	< 0.001	5.279	2.005	13.898	0.001
Nipple retraction (no vs yes)	4.388	2.090	9.210	< 0.001	1.909	0.662	5.508	0.23
Periareolar skin thickening (no vs yes)	6.652	3.308	13.376	< 0.001	1.546	0.543	4.402	0.41
TNE within 2 cm of the NAC (no vs yes)	19.424	8.504	44.366	< 0.001	4.184	1.260	13.898	0.02
Morphology of the lesion (only mass vs including NME)	4.521	2.395	8.536	< 0.001	1.089	0.352	3.367	0.88
TND (> 1 cm vs ≤ 1 cm)	7.189	3.675	14.066	< 0.001	1.482	0.546	4.026	0.44
Overall size (≤ 2 cm vs > 2 cm)	7.305	2.399	22.246	< 0.001	1.096	0.205	5.871	0.92
Suspicious calcification within 2 cm (no vs yes)	3.448	1.787	6.654	< 0.001	0.752	0.289	1.957	0.56

*HER-2* human epidermal growth factor receptor-2, *NAC* nipple–areolar complex, *TNE* tumor–nipple enhancement, *NME* non-mass enhancement, *TND* tumor–nipple distance, *CI* confidence interval, *OR* odds ratio

Among the 175 cases, 88 cases showed asymmetric NAC enhancement, of which 69 were pathologically confirmed to have NAC involvement. The sensitivity, specificity, PPV, NPV, accuracy, and AUC of asymmetric NAC enhancement were 77.53% (69/89), 77.91% (67/86), 78.41% (69/88), 77.01% (67/87), 77.71% (136/175), and 0.777, respectively. Of the 107 cases with TNE within 2 cm of the NAC, 82 showed TNE extending to the NAC, and 25 did not. A total of 70 cases (65 cases in the extending group and 15 in the non-extending group) were pathologically confirmed to have NAC involvement. The sensitivity of TNE within 2 cm of the NAC was as high as 89.89% (80/89), with a specificity of 68.60% (59/86), an NPV of up to 86.76% (59/68), and an AUC of 0.792. The PPV of the TNE extending to the NAC was higher than that non-extending to the NAC (79.27% (65/82) vs 60.00% (15/25), respectively). When both asymmetric NAC enhancement and TNE extending to the NAC were present, the PPV was the highest at 82.35% (56/68) (Table 4).

**Table 4:** Diagnostic performances of asymmetric NAC enhancement and TNE within 2 cm of the NAC, which predict NAC involvement in breast cancer

Predicting factors	Sensitivity	Specificity	PPV	NPV	Accuracy	AUC
Asymmetric NAC enhancement	77.53% (69/89)	77.91% (67/86)	78.41% (69/88)	77.01% (67/87)	77.71% (136/175)	0.777
TNE within 2 cm of the NAC	89.89% (80/89)	68.60% (59/86)	74.77% (80/107)	86.76% (59/68)	79.43% (139/175)	0.792
TNE extending to the NAC	73.03% (65/89)	80.23% (69/86)	79.27% (65/82)	74.19% (69/93)	76.57% (134/175)	0.766
TNE non-extending to the NAC but within 2 cm	62.50% (15/24)	85.51% (59/69)	60.00% (15/25)	86.76% (59/68)	79.57% (74/93)	0.740
Asymmetric NAC enhancement + TNE within 2 cm of the NAC	71.91% (64/89)	82.56% (71/86)	81.01% (64/79)	73.96% (71/96)	77.14% (135/175)	0.772
Asymmetric NAC enhancement + TNE extending to the NAC	62.92% (56/89)	86.05% (74/86)	82.35% (56/68)	69.16% (74/107)	74.29% (130/175)	0.745
Asymmetric NAC enhancement + TNE non-extending to the NAC but within 2 cm	33.33% (8/24)	95.65% (66/69)	72.73% (8/11)	80.49% (66/82)	79.57% (74/93)	0.645

*NAC* nipple–areolar complex, *TNE* tumor–nipple enhancement, *PPV* positive predictive value, *NPV* negative predictive value, *AUC* area under the curve

## Discussion

In this study, we found that the accuracy of CE-CBBCT for the diagnosis of NAC involvement was 83.52%, with a sensitivity of 96.70%, specificity of 70.33%, PPV of 76.52%, and NPV of 95.52%. These data indicate that CBBCT is a reliable imaging approach for predicting NAC involvement before surgery. Furthermore, we found that in multivariate logistic regression analysis, asymmetric NAC enhancement and TNE within 2 cm of the NAC were strongly significant predictors of NAC involvement. In particular, when asymmetric NAC enhancement and TNE extending to the NAC occurred simultaneously, the PPV was highest (82.35%).

Approximately 14.82% (95/641) of our original study population had pathologically confirmed NAC involvement, which is consistent with the findings of previous studies, with an incidence ranging from 8% to 27.7% [4–9]. In our study, CE-CBBCT showed good performance, with a high accuracy of 83.52%, a high sensitivity of 96.70%, a high NPV of 95.52%, and a PPV of 76.52%. The accuracy of MRI in the diagnosis of NAC involvement was 80.8%-87.4% [9, 15]. Liao et al also suggested that MRI had a low PPV in their study and showed a wide discrepancy among different reported series [9]. This indicates that CE-CBBCT might be an alternative option for predicting NAC involvement to MRI. Additionally, in our research, CE-CBBCT demonstrated a specificity of 70.33% in diagnosing NAC involvement (indicating a 29.67% false-positive rate), whereas the specificity of MRI was 80–90% [9, 10]. This 29.67% false-positive rate implied that some patients diagnosed with NAC involvement by CE-CBBCT might not require nipple excision. To effectively mitigate this risk, we suggest utilizing intraoperative frozen biopsies or preoperative biopsy of suspicious lesions for further confirmation, thereby sparing patients from overtreatment. Notably, previous research has already explored CBBCT-guided biopsy, and demonstrated that it was safe and feasible [27]. Therefore, patients who were predicted to have NAC involvement on CBBCT are suggested to undergo preoperative CBBCT-guided biopsy to obtain pathological results of suspicious NAC-involved lesions identified by CBBCT. By combining imaging results with biopsy pathology, clinicians can devise more precise surgical plans for patients.

Univariate analysis in our study showed that many factors were statistically associated with NAC involvement, and previous studies reported similar results [4–6, 15, 17, 19, 32, 33]. However, our multivariate analysis found that only TNE within 2 cm of the NAC and asymmetric NAC enhancement remained statistically associated with NAC involvement. Consistent with our study, these two factors also remained significant in multivariate analysis in other studies [4, 9]. In our study, TNE within 2 cm of the NAC had the highest sensitivity (89.89%) and the highest NPV (86.76%), while the sensitivity of this feature in MRI was 71.0–79.2%, and the NPV of this feature in MRI ranged from 67.1% to 97.5% [4, 17, 34]. The high sensitivity of this CBBCT feature can help exclude NAC involvement, avoid misdiagnosis, and be used as a relative contraindication for NSM. Furthermore, when TNE was not present within 2 cm of the NAC, there was an 86.76% probability that the patient had no NAC involvement, which is particularly important for avoiding unnecessary surgical intervention. The PPV of TNE extending to the NAC was higher than that of TNE non-extending to the NAC, but within 2 cm (79.27% > 60.00%), which was consistent with the findings of previous studies concerning enhancement between the tumor and the NAC [31, 35]. When we combined asymmetric NAC enhancement with TNE extending to the NAC, the PPV was highest (82.35%), indicating that when these two features co-occurred, the probability of NAC involvement was much higher.

Our multivariate analysis revealed no significant correlation between suspected calcification within 2 cm and NAC involvement, similar to the results reported by Moon et al [18] and Lim et al [13]. However, in our study, among cases with suspected calcification within 2 cm, the number of NAC involvement cases was higher than that of NAC non-involvement cases (*n* = 45 vs *n* = 20), and univariate logistic analysis suggested that there was a significant correlation between suspected calcification within 2 cm and NAC involvement (*p* < 0.001). These findings highlight the need for further investigation of this variable. Although TND was a significant factor in univariate analysis, it was not significantly correlated with NAC involvement in multivariate analysis, contradicting the findings of some reports [4, 7]. However, Piato et al [6] also found that TND was not significantly associated with NAC involvement. We hypothesized that the cutoff value and interactions among variables were likely to cause this discrepancy, and further research is needed.

This study has several limitations. First, selection bias is possible because this was a retrospective study. Second, this was a single-center study, limiting the findings’ universality. Thirdly, although two readers evaluated imaging with high agreement in this study, a multicenter study with more readers should be conducted as a follow-up to make the results more reliable and reproducible. Additionally, the interpretation of imaging features significantly relies on the experience of radiologists and might be influenced by subjective factors, which is an inherent limitation of all qualitative studies. We have minimized this impact through pre-evaluation training and increasing the number of radiologists on our study, and we anticipate leveraging artificial intelligence technology in the future to develop more precise and objective predictive models. Finally, CE-CBBCT radiation exposure carries certain risks with mean doses ranging from 11.7 mGy to 16 mGy, and further exploration and development in CBBCT technologies, such as iterative reconstruction, are needed to investigate reducing radiation dose [21, 23].

In conclusion, with comparably high accuracy, CE-CBBCT is a safe and non-invasive modality for the preoperative diagnosis of NAC involvement. Asymmetric NAC enhancement and TNE within 2 cm of the NAC showed good performance in predicting NAC involvement.

## Supplementary information

ELECTRONIC SUPPLEMENTARY MATERIAL

## Abbreviations

AUC: Area under the curve
CE-CBBCT: Contrast-enhanced cone beam breast CT
CI: 95% Confidence interval
ER: Estrogen receptor
HER-2: Human epidermal growth factor receptor
IQR: Interquartile range
NAC: Nipple–areola complex
NPV: Negative predictive value
NSM: Nipple-sparing mastectomy
OR: Odds ratio
PPV: Positive predictive value
PR: Progesterone receptor
ROC: Receiver operating characteristic
TND: Tumor–nipple distance
TNE: Tumor–nipple enhancement
