# Supplementary material for: Performance of contrast-enhanced cone-beam breast CT to predict nipple–areolar complex involvement in early-stage breast cancer
Source: Eur Radiol. 2025 Jul 1;36(1):45–54. doi: 10.1007/s00330-025-11787-8 (PMC12712108; doi:10.1007/s00330-025-11787-8)
Supplement: Supplementary file 1 — ELECTRONIC SUPPLEMENTARY MATERIAL [file 330_2025_11787_MOESM1_ESM.pdf]

# Performance of contrast-enhanced cone-beam breast CT to predict nipple-areolar complex involvement in early-stage breast cancer

# ELECTRONIC SUPPLEMENTARY MATERIAL

Clinicopathologic factors and radiologic features in CBBCT images in patients with or without NAC involvement based on pathological diagnosis

| | | Pathological NAC involvement | | P value |
|---|---|---|---|---|
| | | non-involvement (N=91) | involvement (N=91) | |
| Clinicopathologic factors | | | | |
| Age | | | | 0.07 |
| | ≤50 | 56(61.5%) | 44(48.4%) | |
| | ＞50 | 35(38.5%) | 47(51.6%) | |
| Menopausal status | | | | 0.07 |
| | Postmenopausal | 36(39.6%) | 48(52.7%) | |
| | Premenopausal | 55(60.4%) | 43(47.3%) | |
| Histologic subtype | | | | 0.82 |
| | DCIS | 10(11.0%) | 11(12.1%) | |
| | IC | 81(89.0%) | 80(87.9%) | |
| Nuclear grade | | | | 0.08 |
| | 1 | 0(0.0%) | 1(1.1%) | |
| | 2 | 52(57.1%) | 39(42.9%) | |
| | 3 | 39(42.9%) | 51(56.0%) | |
| Stage | | | | 0.06 |
| | 0 | 10(11.0%) | 11(12.1%) | |
| | 1 | 30(33.0%) | 28(30.8%) | |
| | 2 | 35(38.5%) | 22(24.2%) | |
| | 3 | 16(17.6%) | 30(33.0%) | |
| Postoperative T stage | | | | 0.99 |
| | Tis | 10(11.0%) | 11(12.1%) | |
| | 1 | 38(41.8%) | 39(42.9%) | |
| | 2 | 37(40.7%) | 36(39.6%) | |
| | 3 | 3(3.3%) | 2(2.2%) | |
| | 4 | 3(3.3%) | 3(3.3%) | |
| Postoperative N stage | | | | 0.02 |
| | 0 | 56(61.5%) | 44(48.4%) | |
| | 1 | 24(26.4%) | 18(19.8%) | |
| | 2 | 5(5.5%) | 14(15.4%) | |

| | | | | |
|---|---|---|---|---|
| | 3 | 6(6.6%) | 15(16.5%) | |
| ER | | | | 0.40 |
| | Negative | 22(24.2%) | 27(29.7%) | |
| | Positive | 69(75.8%) | 64(70.3%) | |
| PR | | | | 0.54 |
| | Negative | 30(33.0%) | 34(37.4%) | |
| | Positive | 61(67.0%) | 57(62.6%) | |
| HER-2 status | | | | 0.007 |
| | Not amplified | 59(64.8%) | 41(45.1%) | |
| | Amplified | 32(35.2%) | 50(54.9%) | |
| Ki-67 expression | | | | 0.45 |
| | ＜15% | 19(20.9%) | 15(16.5%) | |
| | ≥15% | 72(79.1%) | 76(83.5%) | |
| Molecular subtype | | | | 0.06 |
| | TNBC | 14(15.4%) | 6(6.6%) | |
| | Luminal A | 16(17.6%) | 10(11.0%) | |
| | Luminal B (HER-2 negative) | 29(31.9%) | 25(27.5%) | |
| | Luminal B (HER-2 positive) | 19(20.9%) | 27((29.7%) | |
| | HER-2 overexpression | 13(14.3%) | 23(25.3%) | |
| Lymphovascular invasion | | | | 0.88 |
| | Negative | 60(65.9%) | 59(64.8%) | |
| | Positive | 31(34.1%) | 32(35.2%) | |
| Radiologic features | | | | |
| Asymmetric NAC enhancement* | | | | ＜ 0.001 |
| | No | 67(77.9%) | 20(22.5%) | |
| | Yes | 19(22.1%) | 69(77.5%) | |
| Nipple retraction | | | | ＜ 0.001 |
| | No | 79(86.8%) | 52(57.1%) | |
| | Yes | 12(13.2%) | 39(42.9%) | |
| Periareolar skin thickening | | | | ＜ 0.001 |
| | No | 76(83.5%) | 37(40.7%) | |
| | Yes | 15(16.5%) | 54(59.3%) | |
| Suspicious calcification within 2cm | | | | ＜ 0.001 |
| | No | 71(78.0%) | 46(50.5%) | |
| | Yes | 20(22.0%) | 45(49.5%) | |
| Multicentric/multifocal lesion | | | | ＜ 0.001 |
| | No | 45(49.5%) | 12(13.2%) | |

| | | | | |
|---|---|---|---|---|
| | Yes | 46(50.5%) | 79(86.8%) | |
| Tumor location | | | | 0.23 |
| | Central area only | 4(4.4%) | 8(8.8%) | |
| | Involving peripheral area | 87(95.6%) | 83(91.2%) | |
| Overallsize | | | | ＜0.001 |
| | ≤2cm | 25(27.5%) | 4(4.4%) | |
| | ＞2cm | 66(72.5%) | 87(95.6%) | |
| Largest diameter | | | | 0.11 |
| | ≤2cm | 32(35.2%) | 22(24.2%) | |
| | ＞2cm | 59(64.8%) | 69(75.8%) | |
| TND | | | | ＜0.001 |
| | ＞1cm | 71(78.0%) | 30(33.0%) | |
| | ≤1cm | 20(22.0%) | 61(67.0%) | |
| Morphology of the lesion | | | | ＜0.001 |
| | Only mass | 63(69.2%) | 30(33.0%) | |
| | Including NME | 28(30.8%) | 61(67.0%) | |
| TNE within 2cm of the NAC | | | | ＜0.001 |
| | No | 63(69.2%) | 9(9.9%) | |
| | TNE extending to the NAC | 17(18.7%) | 67(73.6%) | |
| | TNE non-extending to the NAC but within 2cm | 11(12.1%) | 15(16.5%) | |

HER-2 amplification was defined as 3 + using immunohistochemistry or gene amplification by in situ hybridization.

NAC, nipple-areolar complex; DCIS, ductal carcinoma in situ; IC, invasive carcinoma; ER, estrogen receptor; PR progesterone receptor; HER-2, human epidermal growth factor receptor-2; TNBC, triple negative breast cancer; TND tumor-nipple distance; NME, non-mass enhancement; TNE, tumor-nipple enhancement.

*Missing values.
